# Heme Iron Content in Lamb Meat Is Differentially Altered upon Boiling, Grilling, or Frying as Assessed by Four Distinct Analytical Methods

**DOI:** 10.1155/2013/374030

**Published:** 2013-05-08

**Authors:** Azin Pourkhalili, Maryam Mirlohi, Ebrahim Rahimi

**Affiliations:** ^1^Department of Food Science and Technology, Faculty of Agriculture, Islamic Azad University, Shahrekord Branch, Shahrekord 88137-33395, Iran; ^2^Food Security Research Center, School of Nutrition and Food Science, Isfahan University of Medical Science, Isfahan 8174-73461, Iran

## Abstract

Lamb meat is regarded as an important source of highly bioavailable iron (heme iron) in the Iranians diet. The main objective of this study is to evaluate the effect of traditional cooking methods on the iron changes in lamb meat. Four published experimental methods for the determination of heme iron were assessed analytically and statistically. Samples were selected from lambs' loin. Standard methods (AOAC) were used for proximate analysis. For measuring heme iron, the results of four experimental methods were compared regarding their compliance to Ferrozine method which was used for the determination of nonheme iron. Among three cooking methods, the lowest total iron and heme iron were found in boiling method. The heme iron proportions to the total iron in raw, boiled lamb meat and grilled, were counted as 65.70%, 67.75%, and 76.01%, receptively. Measuring the heme iron, the comparison of the methods in use showed that the method in which heme extraction solution was composed of 90% acetone, 18% water, and 2% hydrochloric acid was more appropriate and more correlated with the heme iron content calculated by the difference between total iron and nonheme iron.

## 1. Introduction

Being a rich source of protein as well as having balanced component of most essential elements like vitamins and minerals, red meat plays an important role in human' diet especially in developing countries where the intakes of synthetic supplements and alternative fortified foods are less in diet. The importance of meat iron depends on its heme iron content with more bioavailability (from 15% to 35%) than that of the nonheme (2% to 20%) [1]. Based on official data reported in Iran, Iran is the sixth world's producer of lamb or mutton meat; annual consumption of red meat is estimated to be 12.3 kg per capita. Lamb meat is the most popular type of meat among Iranians. However, endemic anemia due to iron deficiency is highly prevalent [2].

It is proven that during cooking, as a heat-induced interaction, oxidation of myoglobin results in oxidative cleavage of porphyrin ring of heme, and some portions of heme iron are converted in to nonheme iron. However, studies have shown that the severity of the reaction is influenced by the applied cooking method as well as the type of meat. In this regard, the previous studies have not supported a similar result.

Turhan et al. studied the effect of cooking methods on total and heme iron contents of fish species. They showed significant differences between the cooking methods; the highest total and heme iron losses were observed in grilled fish [3]. While, according to Schricher and Miller, the highest heme iron loss was found in microwave-cooked and baked beef samples [4].

To determine the content of heme iron and nonheme iron in meat, one of them is usually measured and subtracted from the total iron in order to obtain the content of the another one. The measuring methods of heme iron and nonheme iron are performed in a completely different manner this may lead to different interpretations which result in variation among the reported studies. For heme iron measurement, the spectrophotometric method introduced by Hornsey is well referred in the literature [5]. However, there are some slight differences in the adopted experiments conducted by other researchers regarding the proportion of the samples to heme iron extraction solution as well as the composition of the solution used for heme iron extraction with respect to acetone, water, and hydrochloric acid. Variation in the applied methods might also be an alternative source of inconsistency among the results of previous studies.

Since the traditional ways of cooking lamb in Iran are boiling, frying, and grilling, the main objective of this study was to evaluate the effect of traditional cooking methods on the heme iron to nonheme iron conversion. In this regard, this study looked to the correlation between the results of four heme iron measurement methods and the results of its calculation based on non heme iron measurement. 

## 2. Material and Methods

### 2.1. Sample Procedures

Five raw nonprepacked meat samples from sheep (Lori-Bakhtiari, 6 months) were purchased from selected butchery in Isfahan, Iran. Sections of longissimus dorsi meat from each sample were aseptically removed and placed in separate sterile plastic bags to prevent spilling and cross-contamination and were immediately transported to the laboratory in a cooler with ice packs. Muscle samples were trimmed of connective and adipose tissues and sliced into square cuts of 2.5 × 2.5 cm of thickness. The cuts were vacuum packed in four layers of polyethylene packagings and were stored frozen at −18 until analysis. Frozen samples were thawed 4-5 h at 4 ± 2°C before analysis. All the analyses were done in triplicate for each meat sample.

### 2.2. Cooking Methods

The meat samples were subjected to each of the cooking method (boiling, frying, and grilling), while the raw meat was sampled directly as an uncooked control. Internal temperatures were monitored using thermometer (thermometer ST-131 waterproof digital).

Boiling was performed at approximately 97°C (water temperature) for 90 min in stainless steel pan. The internal temperature during boiling was 93°C. The meat samples were pan fried in sunflower oil for 20 min. The internal temperature during frying was determined as 85°C. Grilled meats were prepared using burning stove. The cuts were placed at 10 cm above the flame for 10 min, turned by 2 min interval. The internal temperature was not exceeded at 86°C.

### 2.3. Proximate Composition

Proximate composition of raw and cooked lamb meat was done for moisture, fat, ash, protein, and loss weight content. Moisture contents of ground meat sample were determined by drying in an oven at 105°C until constant weight [6]. The fat content was determined by Folch method [7]. Ash content was determined as dried for 4 h at 125°C at heated temperature oven (500–550°C) for 6–8 h [8]. The protein was determined by micro-Kjeldahl procedure [9]. Loss weight was determined by weighting samples before and after cooking.

### 2.4. Total Iron

An accurately weighed 3 g sample was dried for 4 h at 125°C as heated temperature oven (500–550°C) for 6–8 h. The ash was digested in 5 mL of 2 M HNO_3_ by boiling for about 2 min and then left to cool down to the room temperature. The cooled solution was filtered through Whatman filter paper (no.41) and made up to 25 mL with 2 M HNO_3_. The samples were then analyzed for total iron by atomic absorption spectrophotometery (Model 2380, PerkinElmer, USA) at a wavelength of 248.3 nm [8]. 

### 2.5. Heme Iron

Heme iron was determined using four Hornsey modified methods [5].

*Experiment A. *Ground sample (10 g) was weighed into 50 mL centrifuge tubes. Then, 20 mL of acid-acetone mixture was added (40 mL of acetone, 9 mL of water, and 1 mL of concentrated hydrochloric acid). Each sample was homogenized for 30 s. Then, an additional 20 mL of acid-acetone mixture was added, and the samples were mixed thoroughly the tubes were then capped tightly and kept in the dark for 1 h. The extract was centrifuged at 2200 rpm for 10 min. The supernatant was filtered through glass microfiber filters (Whatman GF/A), and the absorbance was measured at 640 nm (Model 6105, Jenway Uv/Vis Spectrophotometer, UK) against a reagent blank [3].

*Experiment B. *Ground sample (10 g) was weighed into 50 mL centrifuge tubes. Then, 45 mL of acid-acetone mixture was added (45 mL of acetone, 4 mL of water, and 0.5 mL of concentrated hydrochloric acid). Each sample was homogenized for 30 s. The samples were mixed thoroughly the tubes were then capped tightly and kept in the dark for 1 h. Further steps were carried out as in experiment 1 [10].

*Experiment C. *Ground sample (5 g) was weighed into 50 mL centrifuge tubes. Then, 10 mL of acid-acetone mixture was added (40 mL of acetone, 8 mL of water, and 1 mL of concentrated hydrochloric acid). Each sample was homogenized for 30 s. Then, an additional 10 mL of acid-acetone mixture was added, and the samples were mixed thoroughly the tubes were then capped tightly and kept in the dark for 30 min. The extract was centrifuged at 3000 rpm for 20 min, and the supernatant was processed as in experiments 1 and 2 [11].

*Experiment D. *Freeze-dried meat sample (0.05 g) was weighted into 50 mL centrifuge tubes. Then, 20 mL of acid-acetone mixture was added (15.6 mL of acetone, 3.75 mL of water, and 0.65 mL of concentrated hydrochloric acid). Each sample was homogenized for 30 s. The extract was centrifuged at 2200 rpm for 10 min. the supernatant was processed as above in experiments 1 and 2 [12].

The absorbance was multiplied by 6800 and then divided by the sample weight to give the concentration of total pigments in the meat as *μ*g hematin/g meat. The iron content was calculated with the factor of 0.0882 *μ*g iron/*μ*g hematin [3].

### 2.6. Nonheme Iron

Nonheme iron was analyzed by the Ferrozine method described by Ahn et al. [13]. Briefly, 0.50 g of freeze-dried sample was dissolved in 3 mL of 0.1 M citrate phosphate buffer (pH 5.5) and 1 mL of 2% ascorbic acid in 0.2 M HCl and was left to stand at room temperature for 15 min before adding 2 mL of 11.3% trichloroacetic acid and then was centrifuged at 3000 rpm for 10 min. To 2 mL of the supernatant, 0.8 mL of 10% ammonium acetate and 0.2 mL Ferrozine reagent were added, and the absorbance was measured at 562 nm (Model 6105, Jenway Uv/Vis Spectrophotometer, UK) against a standard curve [14]. Using 1000 mg/L stock solution of FeCl_3_, standard solutions were adjusted at 10, 25, 50, and 100 mg/L.

Standard curve was prepared by plotting the absorbance against the several concentrations of FeCl_3_ standard solutions. The represented equation of *y* = 0.0015*x*+ was obtained with *r* ^2^ = 0.92.

All the chemical reagents was from (Merck-Germany) with the exception of Ferrozine reagent which was from (Sigma-USA).

### 2.7. Statistical Analysis

All data were tested using one-way ANOVA test and the pairwise comparison was performed using Tukey-Kramer multiple comparison test. The level of significance was established at *P* = 0.05. Spearman correlation was used to find the most correlated heme measuring method to non heme iron determination Ferrozin method. SPSS software was used for data analyzing.

## 3. Result and Discussion 

The results of the proximate analyses are presented in Table 1. The highest and the lowest moisture losses were found to be 20% and 9% in the fried and grilled samples, respectively. Fat content increased in fried and grilled samples and decreased in boiled ones. Previously, it was reported that the lowest moisture loss happens through grilling [15]. Frying and boiling resulted in the highest and the lowest fatty material in the cooked lamb pieces, respectively, which could be explained by oil absorption in fried samples [16, 17].

During the cooking process, meat protein denaturation occurs, and subsequent reduction in water holding capacity of proteins causes moisture loss and an increase in total dry matter and yields higher concentration of other components in the cooked meat. However, a portion of fat is melted and runs out with cooking juice; hence, the remained fatty material in the cooked meat is the outcome of the both above processes which completely depends on the cooking method. The ash content increased by grilling and frying up to 2.91% and 16.71% from that of the ash in raw sample, respectively, but it was reduced by 51.2% in the boiled samples [18].

The results of total iron, nonheme iron and heme iron determination are presented in Table 2, where heme iron was measured using four tested experiments, all derived from the Hornsey method [5]. Total iron was decreased after cooking by 52.28%, 33.37%, and 30.44% of its primary concentration in raw sample by boiling, frying, and grilling, respectively. The difference between raw, boiled, and fried samples was significant (*P* < 0.05). The least total iron change occurred in grilled samples. Instead, boiling caused noticeable iron loss. Although the decrease in total iron due to cooking was previously reported by others, the result obtained in the present study was in contrast to the result found by Turhan et al. in which the highest total iron loss happened in grilling (52.60%) [3]. Such difference may be due to different type of examined meats (fish and lamb) as well as different time and temperature profile applied in that study.

Reporting the iron concentration on the wet base gave rise to different results. An increase in the total iron in some of the cooked samples was observed (data is not shown). Such increase could be explained by decrease in the moisture content of the cooked samples due to protein denaturation and reduction in water holding capacity [12]. It was shown that cooking (baking) caused an increase in the total iron content in beef and lamb meat by 73% and 43%, respectively. Likewise, similar results were reported for beef by Purchas et al. when wet-based changes took in to account [14].

Nonheme iron also decreased after heat treatment (Table 2) in all the examined samples. Since the total iron underwent basic changes, variation in nonheme iron can be better explained by its ratio to the total iron in each treated sample which is presented in Table 3. In that setting, the highest non heme iron was observed in boiled ones. Because during a boiling treatment the temperature does not exceed 100°C, it is considered as mild cooking method, but long processing time (90 minutes) in our study alongside water vapor pressure may induce more non heme formation in boiled samples than that of fried and grilled counterparts.

Increase in the non heme iron concentration after cooking was reported by others [4, 14].

The presented results of heme iron determination in Table 2 showed that the slight differences in the four Hornsey derived heme iron measuring methods examined in this study have great impact on the obtained results. Using method D, the determined heme iron concentration exceeded the total iron content of the given samples. Using method C, the same result was observed. Bi variable Spearman correlation between the results of each heme iron measuring method and the calculated heme iron after non heme iron subtraction from that of the total iron was performed. The results showed that, using methods A and B, there was a significant correlation between the two sets of heme iron data mentioned above (*P* = 0.003 and *P* = 0.014). But, it was not the case for the two other methods (C and D). For the methods tested, A, B, C, and D, *r* ^2^ was obtained as 0.997, 0.986, 0.9, and 0.5, respectively. Therefore, comparing to other examined heme iron measuring methods, the method adopted by Turhan et al. in which heme extraction solution included more water and less acetone gave better results. Also, it was found in the present study that the experiments time length reduction had great impact on the accuracy of the results. This was considered in the given experiment [3].

Pigmentation and subsequent turbidity of the heme containing extraction solution is the main source of error for its spectrophotometric measurement. The higher proportion of acetone to other components of the extraction solution adversely affected the stability of the given solution. Therefore, the less acetone content in the heme extraction solution in method A could be regarded as one of the reasons that this method resulted in less pigmentation and provided more stable results comparing to other tested experiments.

 Despite the sufficient number of repeated measurements, coefficients of variations obtained in the spectrophotometric measurements of heme iron by all tested methods analytically were not considered as proper values, which indicate a vast variation among the repeated measurements. It was particularly observable in the cooked meat samples and was more troublesome for the samples in which heat-induced Millard reaction and caramelization developed more complex colored substances.

Using the four experiments, boiled samples persistently showed to have the least heme iron content (Table 2). Based on the both reliable methods (A and B) in this study, heme iron concentration decreased in the all heat-treated samples; however, significant reduction occurred in boiling (*P* > 0.05). This is in accordance with the result of Turhan et al. [3] that stated that heme iron in fish reduced to 40.06%, 27.87%, 54.45% and 69.70% in baking, grilling, microwave, and boiling, respectively. In another study, it was illustrated that the heme iron content of meat decreased after heat treatment [19]. But, increase in the heme iron concentration through cooking (on the wet bases) was reported by about 53.48%, 33.92%, and 61.93% of the raw sample's heme iron concentration for beef, lamb, and turkey meat, respectively [12].

In Table 3, proportions of heme iron and non heme iron to the total iron in each set of samples are shown. Here, heme iron was obtained by method A. Nonheme iron was separately measured, using Ferrozine method. A logical adverse trend is observable between the presented data which confirms the primary result of this study stating that method A can be regarded as the most reliable and reproducible mean of heme iron spectrophotometric determination.

## 4. Conclusion

The results of this study showed that among the traditional cooking methods currently employed in Iran, grilling has the lowest impact on the total iron reduction and heme iron to nonheme iron conversion in lamb meat. In contrast, boiling has the most deteriorative effect on the nutritional value of lamb meat regarding the iron content and the relevant changes. This reduction could be critical when iron bioavailability is of concern in a society. Moreover, comparing the results derived from Hornsey's heme iron determination method, the one modified by Turhan et al., would bring about more accurate results which are well correlated with the results of Ferrozin nonheme iron method.

## Figures and Tables

**Table 1:** Proximate composition of raw and cooked lamb (dry weight basis).

	Moisture (%)	Fat (%)	Ash (%)	Protein (%)	Loss weight (%)
Raw	73.60 ± 0.28*	33.51 ± 0.31	3.77 ± 0.03	63.40 ± 0.21	—
Boiled	60.50 ± 0.09	25.64 ± 0.74	1.84 ± 0.04	53.84 ± 0.48	46.80 ± 0.23
Fried	53.33 ± 2.25	58.10 ± 1.15	4.40 ± 0.01	59.45 ± 0.33	42.42 ± 0.45
Grilled	64.16 ± 0.75	34.16 ± 0.45	3.88 ± 0.04	60.05 ± 0.38	32.88 ± 1.05

*Data in the table are means of triplicate independent experiments.

**Table 2:** Total iron, nonheme iron, and heme iron, measured by four distinct methods in raw and cooked lamb (*μ*g/g) (dry weight basis).

	Total iron	Nonheme iron	Heme iron							
			A		B		C		D	
Raw	67.91 ± 8.07^a^	21.19 ± 2.89^a^	*44.62 ± 1.38^ad^		38.92 ± 2.49^ad^		73.67 ± 14.19^ad^		73.31 ± 31.4^a^	
			**40.98	49.14	36.44	41.41	60.88	95.02	31.2	99.6
			***6.93		6.38		19.26		42.81	
Boiled	32.41 ± 13.63^b^	17.36 ± 1.68^a^	21.96 ± 1.08^bc^		21.42 ± 6.61^bc^		41.37 ± 7.09^b^		62.67 ± 32.6^a^	
			20.51	23.09	14.91	30.65	30.87	56.51	26.98	85.16
			4.92		30.85		17.13		36.51	
Fried	45.25 ± 6.35^b^	14.98 ± 3.96^a^	32.57 ± 6.39^acd^		31.36 ± 2.00^acd^		65.94 ± 9.62^acd^		82.01 ± 18.48^a^	
			22.91	43.95	28.42	33.40	54.34	80.84	54.57	93.56
			19.63		6.37		14.59		22.53	
Grilled	47.24 ± 16.22^ab^	15.48 ± 4.30^a^	35.91 ± 6.97^ad^		33.74 ± 3.85^ad^		72.19 ± 22.33^acd^		82.0 ± 40.4^a^	
			26.12	48.30	30.38	37.94	44.82	96.87	46.8	125.9
			19.40		11.41		30.95		49.29	

Within the columns, the values with different letter are significantly different (*P* < 0.05).

*Mean ± standard error.

**Minimum−Maximum range.

***Coefficient of variation.

**Table 3:** Percentage of heme iron and nonheme iron to total iron in raw and cooked lamb meat.

	Heme iron (%)*	Nonheme iron (%)**
Raw	65.70	31.20
Boiled	67.75	53.56
Fried	71.97	33.10
Grilled	76.01	32.76

*Heme iron determined by method A.

**Nonheme iron determined by Ferrozine method.
